# Irradiation-induced β to α SiC transformation at low temperature

**DOI:** 10.1038/s41598-017-01395-y

**Published:** 2017-04-26

**Authors:** Chad M. Parish, Takaaki Koyanagi, Sosuke Kondo, Yutai Katoh

**Affiliations:** 1 Oak Ridge National Laboratory, Oak Ridge, TN 37831 USA; 2 Institute of Advanced Energy, Kyoto University, Uji, Kyoto 611-0011 Japan

## Abstract

We observed that β-SiC, neutron irradiated to 9 dpa (displacements per atom) at ≈1440 °C, began transforming to α-SiC, with radiation-induced Frank dislocation loops serving as the apparent nucleation sites. 1440 °C is a far lower temperature than usual β → α phase transformations in SiC. SiC is considered for applications in advanced nuclear systems, as well as for electronic or spintronic applications requiring ion irradiation processing. β-SiC, preferred for nuclear applications, is metastable and undergoes a phase transformation at high temperatures (typically 2000 °C and above). Nuclear reactor concepts are not expected to reach the very high temperatures for thermal transformation. However, our results indicate incipient β → α phase transformation, in the form of small (~5–10 nm) pockets of α-SiC forming in the β matrix. In service transformation could degrade structural stability and fuel integrity for SiC-based materials operated in this regime. However, engineering this transformation deliberately using ion irradiation could enable new electronic applications.

## Introduction

SiC is a major contender for future applications in advanced nuclear energy systems^1–3^, as well as for electronic or spintronic applications requiring ion irradiation processing^4^. The β-variant (3C) of SiC is preferred for structural (and many other) applications due to its isotropy, but β-SiC is metastable^5^ and undergoes a phase transformation at high temperatures (typically 2000 °C and above) to hexagonal or rhombic SiC, collectively called α-SiC, most often 4 H or 6 H polytype^6^. Nuclear fission and fusion concepts envision using SiC in applications such as fission-product barriers in fuel kernels^7^, in cladding for accident-tolerant light-water reactor fuels^8^, and in fusion blanket concepts^9^. β-SiC is well known for excellent radiation tolerance^10, 11^. SiC is a nearly ideal material for extreme-environment applications primarily due its inherently irradiation-tolerant matrix^12^, but also due to a combination of high-temperature strength and creep resistance and resistance to corrosion from harsh coolants such as Pb-Li. The main disadvantage of SiC is its brittleness, but proper composite design of SiC matrix/SiC fiber composites allows for the amelioration of this brittleness^9^; indeed, SiC/SiC matrix turbine hot-section components is going to be commercialized into jet engines for commercial passenger service. SiC/SiC composites retain good mechanical strength and fracture resistance under many irradiation conditions^13–15^. Composites and liquid-phase sintered SiC are both strong candidates for accident-tolerant light-water reactor fuel cladding or matrices^9, 16, 17^, as a response to the detrimental properties of the present zirconium-alloy fuels demonstrated by the Fukushima disaster, although hydrothermal corrosion may be a limiting factor^18–22^. SiC is also an important high-temperature/high-power semiconductor^4, 23^, and ion implantation (and its associated damage) is important for device fabrication.

Of SiC’s >100 known polytypes, the cubic 3C β-SiC polytype is the main choice for structural and nuclear applications^24, 25^, primarily because of lower processing temperatures and higher isotropy (especially irradiation swelling isotropy) compared to the hexagonal or rhombic α-SiC phases. Transformation by thermal annealing of β to α phase SiC has been studied extensively, and is generally found to occur by slow migration of {111}_β_||{0001}_α_ boundaries with fast migration along directions perpendicular to <0001>^26–28^, and progresses only at temperatures in excess of about 1800 °C, and rapidly only above ~2000 °C. A single observation of lower-temperature α→β was reported^29^, with no details. In a predominantly α-phase SiC material, reaction bonded with a small fraction of β-SiC and containing excess Si, the transformation of β → α was observed under neutron irradiation and post-irradiation annealing^30, 31^ (T_irrad_ ≈ 150 °C, T_anneal_ ≈ 1200 °C), but reports of low-temperature (<1800 °C) β → α transformation in high-purity nuclear-grade β-SiC appear lacking.

In the present study, we irradiated high-phase-purity 3C β-SiC, grown by chemical vapor deposition, in the mixed-spectrum High Flux Isotope Reactor (HFIR) with an average irradiation temperature of 1438 ± 100 °C, to a dose of 9 dpa. Previous characterization showed the starting (unirradiated) material contained no α-SiC phase^32^. Transmission electron microscopy (TEM), scanning TEM (STEM), and high-resolution transmission electron microscopy (HRTEM) studies of the post-irradiated material were performed to determine what features had evolved; our 1440 °C is among the highest temperature irradiations performed on high-purity β-SiC, and was intended to allow further insight to the temperature/fluence damage mechanism map (e.g., ref. 24, Fig. 17). By using higher-purity starting material and a state-of-the-art S/TEM instrument, we intended to determine if the mechanism maps were accurate in this relatively unexplored region. Based on the published mechanism maps, at 1440 °C, 9 dpa, we expected to see voids^33, 34^, faulted Frank loops on {111} planes^22^, and possibly unfaulted loops or the early formation of a dislocation network.

## Results

**Figure 1:**
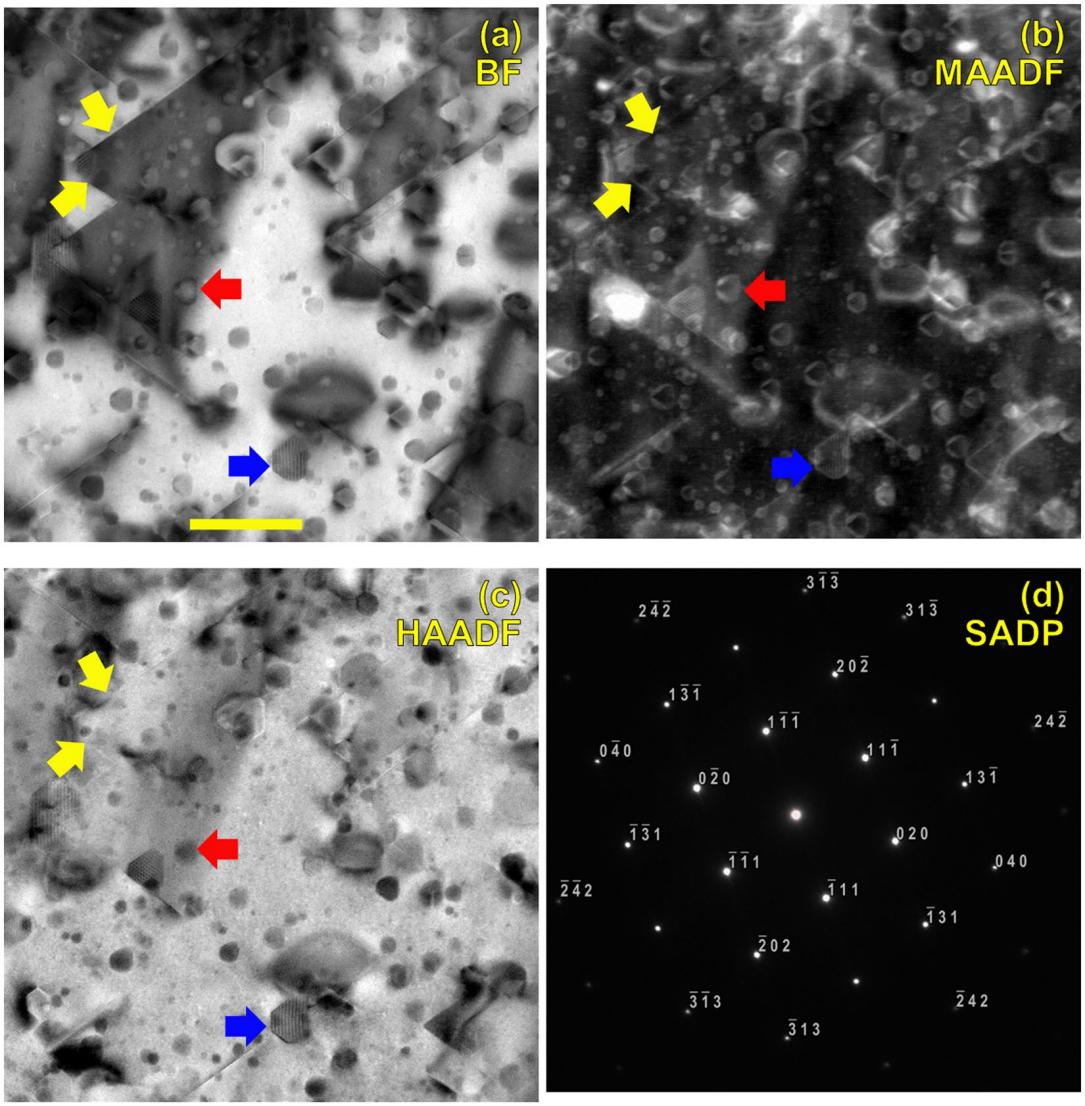
(**a**) Bright field, (**b**) medium-angle annular dark-field, and (**c**) high-angle annular dark field STEM images on the [101] zone axis. (**d**) Selected-area diffraction pattern. Yellow arrows denote 11-1 and -111 Frank loops. Red arrows denote a tetragonal void with strong diffraction contrast. Blue arrow denotes a region of second-phase material. Scale bar: 50 nm.

**Figure 2:**
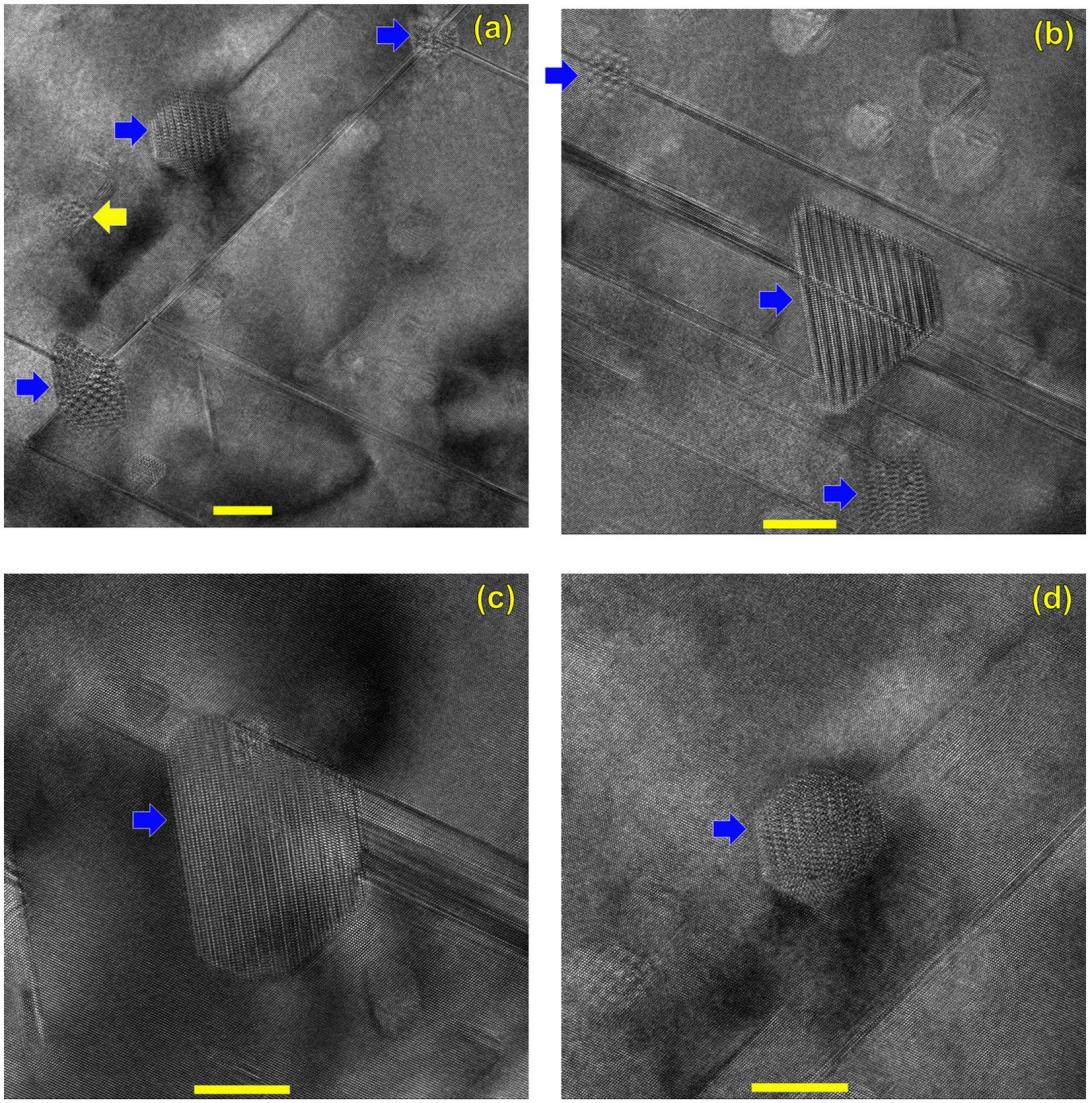
HRTEM images on the [101] zone. Several apparent second-phase precipitates, arrowed, are present. Most or all of these precipitates are located adjacent to a Frank loop (blue ⇒ arrows), and one appears not to be adjacent to a Frank loop (yellow ⇐ arrow). All scale bars 10 nm.

The microstructure after irradiation consists of Frank (faulted) loops on {111} planes (Fig. 1, yellow arrows), tetragonal voids (Fig. 1, red arrow), and small (~5–10 nm) regions that showed contrast inconsistent with the expected microstructural features (Fig. 1, blue arrow). These small regions were not expected from published mechanism maps. These unexpected features were seen to be present in several different shapes, with sizes ~5–10 nm, and nearly all of these features were found to be associated with at least one Frank loop (Fig. 2). Out of the nine pockets of anomalous contrast shown in Fig. 2, eight appear to be in contact with a Frank loop (blue ⇒ arrows) and one does not appear to be in contact with a Frank loop (yellow ⇐ arrow).

**Figure 3:**
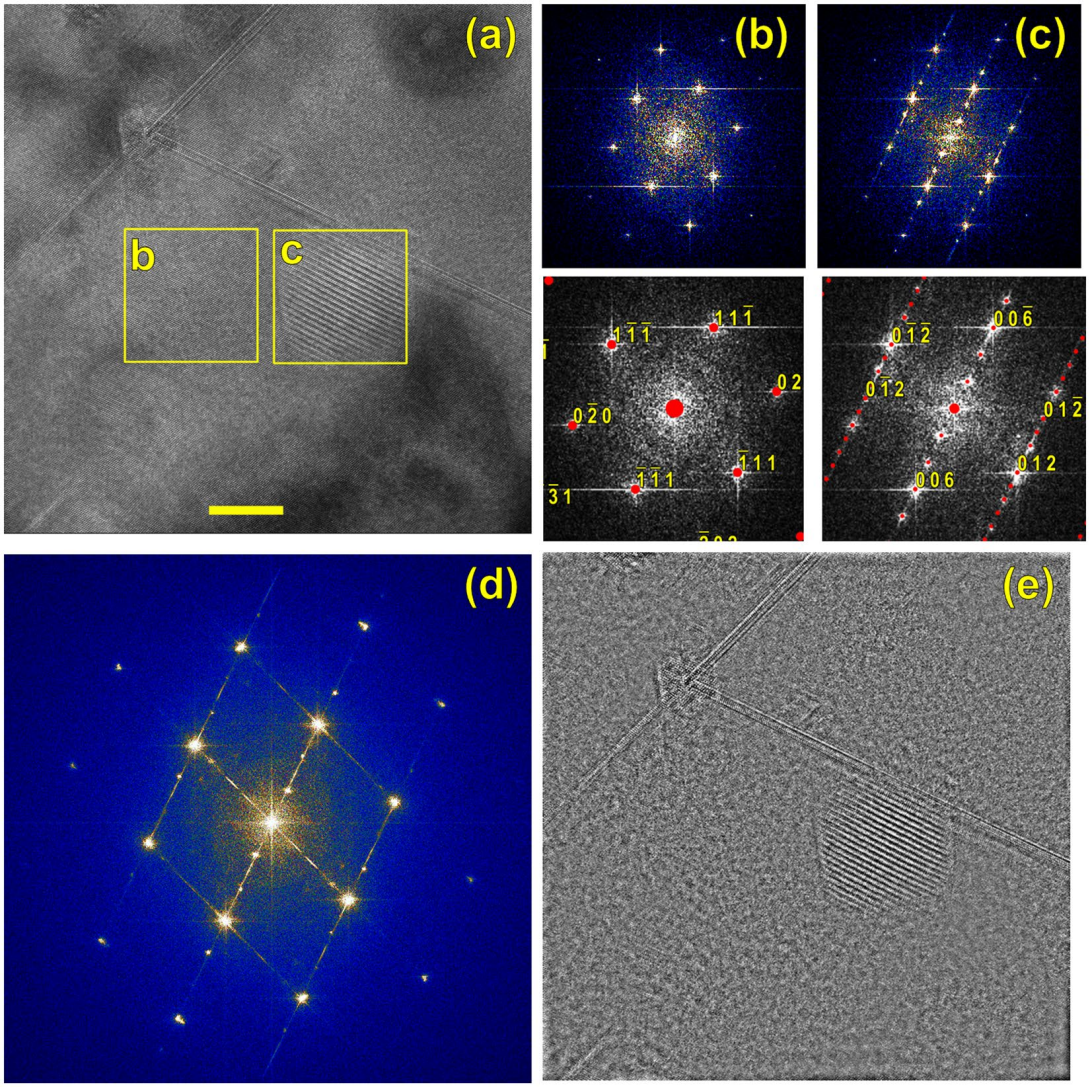
HRTEM image on the [101] matrix zone. Boxes marked “b” and “c” denote the Fourier transforms of (**b**) and (**c**), which also show calculated diffraction patterns for 3C and 6H SiC. (**d**) The Fourier transform of the entire image, showing dislocation streaks, cubic spots, and precipitate spots. (**e**) Inverse Fourier transform after the cubic spots were removed from (**d**); only the precipitates and dislocations remain. Scale bar 10 nm.

Detailed examination of these features indicate that they are α-SiC phase pockets inside the radiation-damaged β-SiC matrix. Regions of matrix and precipitate are marked in Fig. 3(a), and Fourier transforms of these regions are shown in Fig. 3(b,c). The region of 3(b) indexes as cubic β-SiC, on the [101] zone axis, and the Frank loops are seen to lie on the $\pm (11\overline{1})$ and $\pm (\overline{11}1)$ planes. The pocket of second phase indexes as the 6H variant of α-SiC, Fig. 3(c), and the $[0006]$ direction of the 6H phase is aligned perfectly with the $[\overline{11}1]$ direction of the cubic matrix; because the lattice plane spacings d_(111)_ of β-SiC and d_0006_ of 6H α-SiC are both 0.252 nm, this orientation relationship is expected. A Fourier transform of the entire micrograph (Fig. 3(d)) shows the expected complexity: bright β-SiC spots, weak α-SiC spots, and streaking associated with the Frank loops. Filtering out the β-SiC reflections and inverse transformation yields Fig. 3(e), which shows the β-SiC matrix removed and only the defects, such as the α pocket and the Frank loops, remaining.

**Figure 4:**
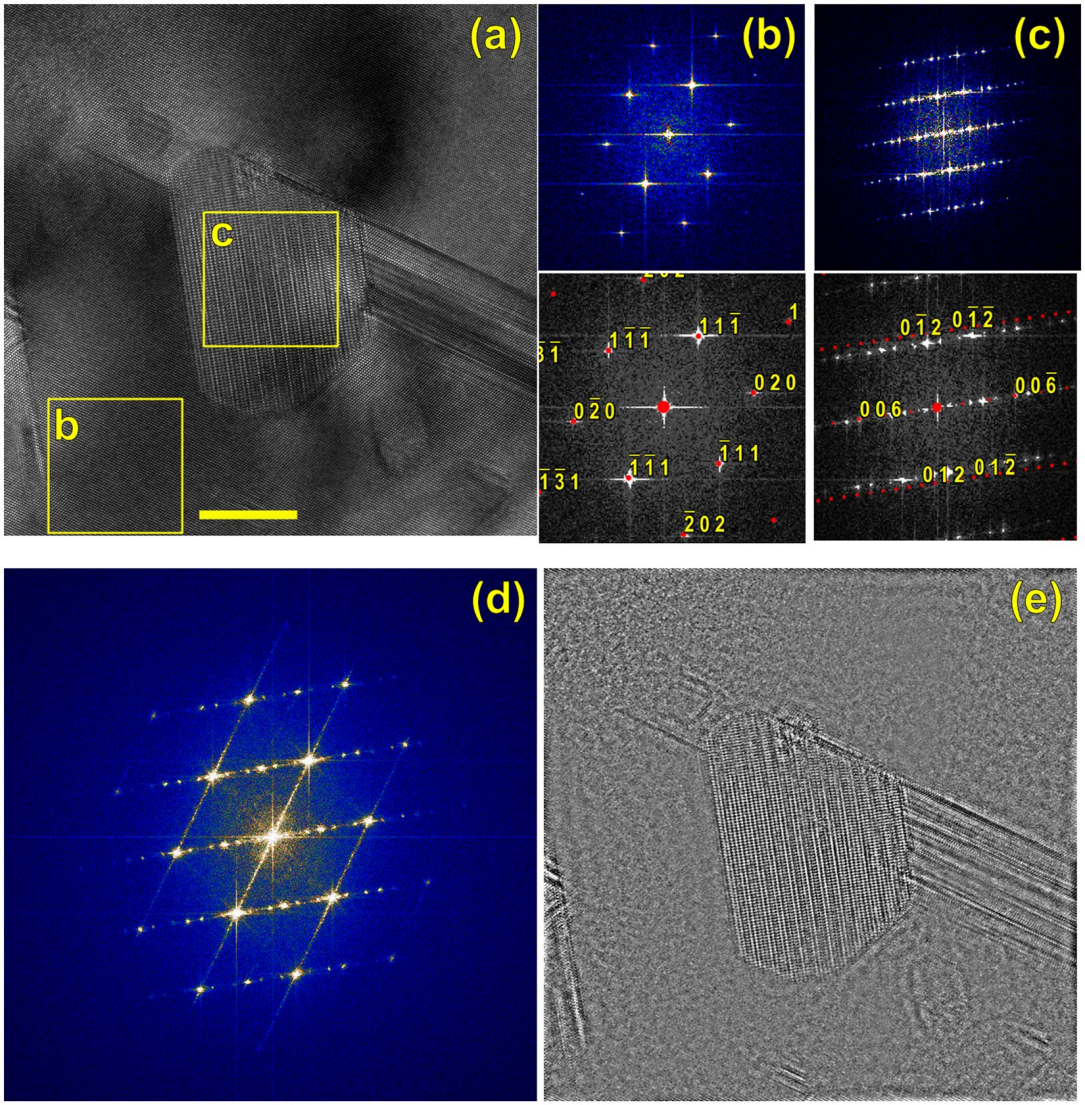
HRTEM image on the [101] matrix zone. Boxes marked “b” and “c” denotes the Fourier transforms of (**b**) and (**c**), which also show calculated diffraction patterns for 3C and 6H SiC. (The 6H pattern is not an exact match). (**d**) The Fourier transform of the entire image, showing dislocation streaks, cubic spots, and precipitate spots. (**e**) Inverse Fourier transform after the cubic spots were removed from (**d**); only the precipitates and dislocations remain. Scale bar 10 nm.

Not all of the α-SiC pockets show the perfect <111>_β_||<000${\mathscr{l}}$>_α_ orientation relationship, Fig. 4. Fourier transforms of matrix and second-phase pocket from Fig. 4(a) are shown in Fig. 4(b,c). The β-SiC matrix indexes identically as before. However, the α-like diffractogram is rotated such that the basal direction is parallel to the <020>_β_ direction of the matrix, which is unexpected. Although a 6H pattern is superimposed on the diffractogram in the bottom of Fig. 4(c), this is not an exact match. The point-to-point spacing in each of the $\{000{\boldsymbol{\ell }}\}$ zones is ~0.6 nm^−1^ (or real-space spacing ~1.6 nm), which is near but not exactly the 6H c-direction lattice parameter. By finding the calibration (nm^−1^/pixel) for the diffractograms from the β-SiC pattern in panel (b), the same magnification can be applied to the 6H pattern superimposed in panel (c). The simulated zones such as ($01\bar{1}\ell$) and ($0\bar{1}1\ell$) in Fig. 4(c) are seen to lie outside the experimental zones, indicating a possible lattice expansion. Regardless, the lack of an exact match is probably due to either strain imposed by not lining up with the easy ${\langle 111\rangle}_{\beta }\parallel {\langle 000\ell \rangle}_{\alpha }$ direction, and/or the α pocket is not necessarily a single-polytype region. (Transformation to multiple α phases is observed during thermal β → α transformation resulting in, essentially, “one dimensional disorder” along <0001>^35^). The entire image Fig. 4(a) was Fourier transformed, filtered to remove β-SiC, and inverse transformed, showing only the Frank loops and second phase particles, Fig. 4(d,e).

**Figure 5:**
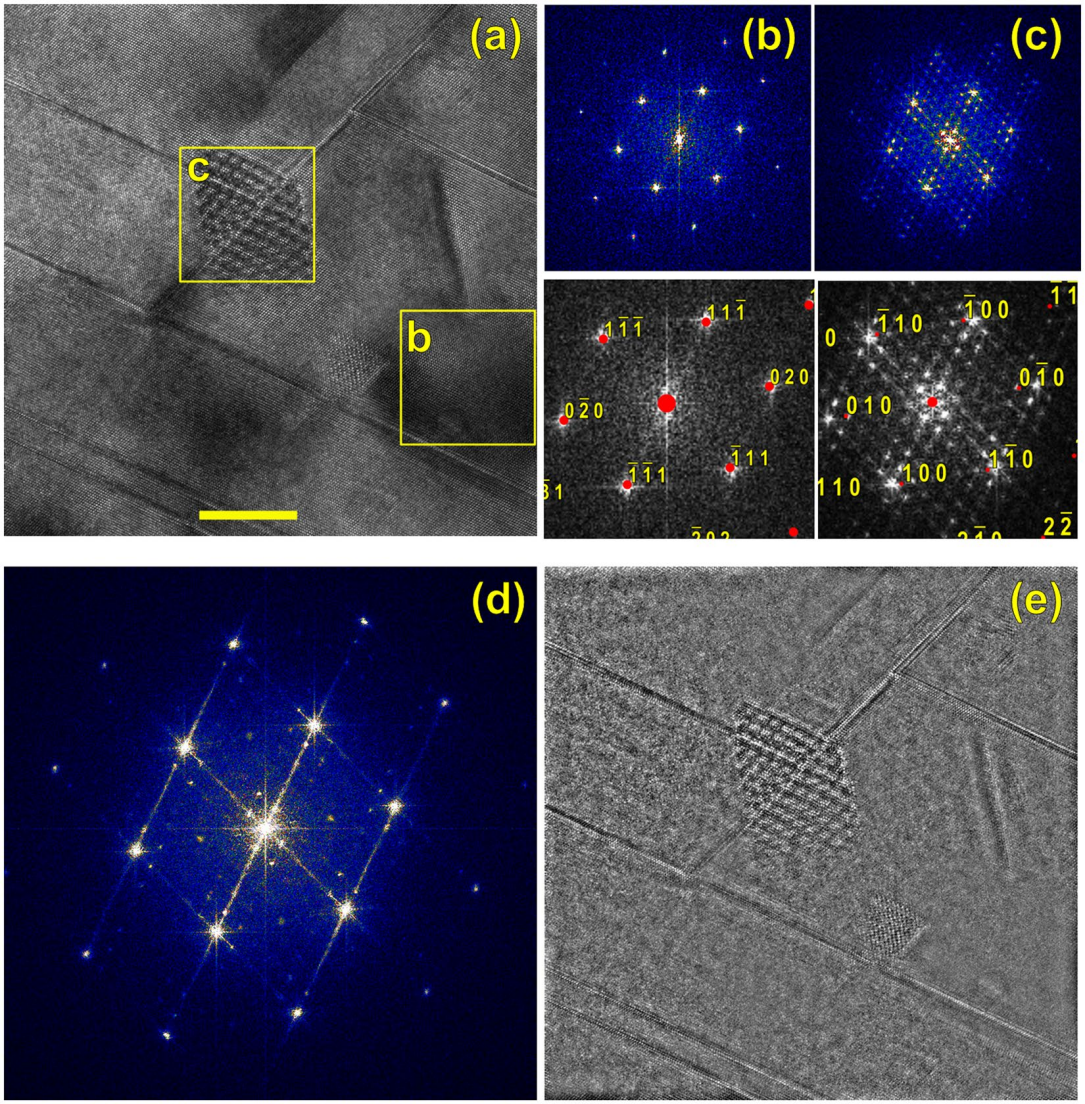
HRTEM image on the [101] matrix zone. Boxes marked “b” and “c” denotes the Fourier transforms of (**b**) and (**c**), which also show calculated diffraction patterns for 3C and 6H SiC. (**d**) The Fourier transform of the entire image, showing dislocation streaks, cubic spots, and precipitate spots. (**e**) Inverse Fourier transform after the cubic spots were removed from (**d**); only the precipitates and dislocations remain. Scale bar 10 nm.

Pockets of α with <101>_β_||<0001>_α_ were also observed. The matrix and α-pocket regions marked in Fig. 5(a) were Fourier transformed into Fig. 5(b,c). Again, the matrix indexes as clean <101>_β_. A more hexagonal or hexagon-like pattern is found for the second-phase pocket, Fig. 5(c). The pattern does not index satisfactorily as, for instance, <0001> −oriented 6H α-SiC (bottom half, Fig. 5(c)). Likely, a double-diffraction condition is occurring in which the α-phase is near the exit surface of the foil, and strong β reflections are causing the moiré-like effects seen in the image. Supplemental Figure S1 shows a double-diffraction simulation where 6H <0001> patterns are centered with the origins on each of the strong β <101> reflections; the match is imperfect but quite close. We therefore conclude that Fig. 5 shows an α-SiC precipitate oriented with <101>_β_||<000*l*>_α_, although the exact α-polytype cannot be discerned, because all <0001>α-SiC diffraction patterns are very similar, showing a hexagon. The 2H, 4H, 6H, 8H, etc. all show the same spacing in the hexagonal pattern, as well. The overall diffractogram (Fig. 5(d) shows the expected features and filtering and inverse transformation leaves only the Frank loops and α pocket, Fig. 5(e).

## Discussion

As illustrated above, we have observed the incipient conversion of high-purity β-SiC into α-SiC, under neutron irradiation, at temperatures (~1440 °C) far below temperatures needed for the thermal conversion of β → α SiC. We have also noted that almost all of the observed pockets of α phase (Fig. 2) are associated with a Frank loop. Because of the nature of TEM, that is, a two-dimensional projection of a three-dimensional thin foil, we cannot say unequivocally that the α pockets were in intimate contact with the Frank loops. (Similarly, the small number of non-Frank-loop associated α could have formed on loops that were milled off during thin foil preparation). However, because the Frank loops are essentially a confined region of α-phase (a stacking fault), and stacking faults nucleate the thermal β → α transformation^26^, we hypothesize that these irradiation-induced Frank loops serve as pre-existing nucleation sites for the α pocket formation in this situation, and reducing the nucleation barrier. Because faulted loops are generally not present in unirradiated material, we can hypothesize they are assisting the β → α transition at lower temperature. However, faulted loops have also been observed in lower temperature SiC irradiations that did not show β → α conversion, so it appears that faulted loops are a necessary but not sufficient condition.

This process appears fundamentally different from some previously observed phenomena in irradiated materials. For instance, growth of second phases in zircon, hafnon, and thorite were observed to occur from the melt zone of a displacement cascade^36^. However, SiC is not believed to produce large clusters from cascades or suffer a thermal-spike melt^37^, so a different mechanism is probably present in SiC compared to zircon, hafnon, and thorite. The graphite to diamond irradiation-induced transformation has also been observed^38, 39^, and attributed to a reduced effective free energy difference under irradiation. Given the small energy difference between SiC polytypes under equilibrium conditions, a reduced effective free energy difference during irradiation seems likely. It is possible that the local increase in point-defect density or the increased local energy state assists growth from the Frank loop edge, using a Frank loop as an α nuclei, to form a region of α phase.

Here we compare, very roughly, thermal diffusion to radiation-enhanced diffusion. A few hours at 2000 °C can transform a large fraction of β-SiC to α-SiC^6, 28^, with corresponding diffusion lengths L (calculated as √(Dt) for diffusion coefficient D^33, 34^ and time t) of L_C_ ≈ 2000 nm and L_Si_ ≈ 200 nm. At 1440 °C for t = 9 × 10^6^ sec (the approximate in-reactor temperature and time), thermal diffusion would produce L_C_ ≈ 60 nm and L_Si_ ≈ 3 nm. Using the SiC radiation enhanced self-diffusion coefficients and point-defect concentrations from ref. 40, we estimate L_C_^RE^~300 nm and L_Si_^RE^~100 nm at 1440 °C for 9 × 10^6^ sec. Although L_C_^RE^ and L_Si_^RE^ numbers are approximate, these radiation-enhanced self-diffusion lengths are roughly comparable to higher temperature thermal conditions that produce partial b to a transformation, suggesting that increased diffusivity arising from radiation-induced point defects allows the transformation to occur in observable periods at lower temperatures observed in this work. This leads to the important hypothesis that higher damage rates, obtainable with ion or electron accelerators, could drive this β to α transformation more quickly and be used in an industrial or microelectronic application.

Polytype nanostructuring of SiC is an emerging field for future electronics and spintronics (for instance, refs 4, 41 and 42). If the presently observed low-temperature phase transformation can be induced via high-energy ion or electron irradiation (which can both produce displacement damage broadly similar to neutrons) then the control of the transformation may potentially be engineered for electronic or spintronic applications.

In summary, we report the conversion of high-purity β-SiC to α-SiC at surprisingly low temperature (~1440 °C) under mixed-spectrum neutron irradiation to ~9 dpa. 6 H polytype α-SiC has been unambiguously identified, and other polytypes may be present. Both the expected <111>_β_||<000*l*>_α_ orientation, and unexpected orientations, e.g., <020>_β_||<000*l*>_α_ and <101>_β_||<000*l*>_α_ have been observed. Regardless of the mechanism, the observation of the β → α conversion in SiC under irradiation may have significant implications for in-service behavior, due to changes in isotropy and increases in boundary surface area that could affect diffusion of fission products^43^. For instance, in TRISO fission fuel, CVD β-SiC serves as a barrier to fission product, fission gas, and actinide escape. Under certain conditions (e.g., refs 44 and 45) the SiC layer in TRISO could experience doses and temperatures similar to those in this study, and conversion of β → α SiC would have effects on the containment of radioisotopes presently not understood or accounted for in TRISO models. Further, under accident conditions, the SiC – which would contain copious Frank loops – would be subjected to high temperatures (perhaps 1700 or 1800 °C), and the pre-existing Frank loops might serve as excellent nucleation sites for the thermal conversion of β → α SiC during the loss-of-coolant temperature excursion. In short, the β → α SiC conversion will have implications for future nuclear applications, and needs to be studied and modelled in detail. Further, different polymorphs can be used in electronic or spintronic engineering^4^, and therefore introduction of these domains in a controlled manner using ions or electrons (rather than neutrons) may lead to new semiconductor or optoelectronic applications.

## Methods

The 3C-SiC samples were chemical vapor deposition samples from Rohm and Haas Advanced Materials (presently Dow Chemical, Woburn, MA). Samples were in the form of disks of size 6 mm diameter and 0.5 or 3.2 mm in thickness. They were irradiated to a fluence of 9.6 × 10^25^ n/m^2^, about 9–10 dpa, in the ORNL High Flux Isotope Reactor, in the METS irradiation campaign. Thermometry indicated an irradiation temperature of approximately 1438 ±100 °C. ±100 °C is a pessimistic bound based on melt wires; we estimate ±25 °C is more likely in reality, based on experience from other irradiation campaigns. Dose rate was ~1 × 10^−6^ dpa/sec.

Samples were prepared by extracting the disks from the irradiation capsules in the ORNL hot cells, shipping them to our hands-on irradiated materials lab (LAMDA)^46^, cleaning, grinding, polishing, and mounting for focused ion beam (FIB) preparation, all in a radiological contamination zone. The samples were then moved from the radiological contamination zone to the clean suite of our laboratory, and imaged in an FEI Versa DualBeam FIB-SEM (scanning electron microscope) tool. Electron backscatter diffraction was used to find grains oriented near <101> in-plane, which were prepared for TEM using the FIB system. Samples were cleaned down to 2 kV Ga^+^ in the Versa DualBeam system, and then were transferred to a Fishione Nanomill system and milled with 900 eV and 600 eV Ar^+^ at ±10° off glancing incidence. This Ar^+^ post-FIB cleaning removes essentially all FIB damage in SiC. By performing the Ar^+^ cleaning less than five minutes prior to loading the samples into the TEM, the possibility of oxidation or anomalous oxide contrast is minimized or eliminated.

TEM and STEM were performed in an FEI Talos F200X tool^47^, operated at 200 keV. Both STEM and TEM were performed with 3C-SiC grains on the <101> orientation. STEM images were acquired in high-angle annular dark field, medium angle annular dark field, and bright field modes. Estimated STEM detector collection angles were: HAADF, 77–200 mr; MAADF, 18–77 mr; BF, 0–10 mr. TEM images were acquired with a large objective aperture, using the 4096 × 4096 pixel TEM camera (FEI Ceta) mounted to the Talos tool. Post processing used Gatan Digital Micrograph software. The double diffraction pattern (Supplemental Figure S1) was produced by simulating 3C and 6H patterns in CrystalMaker^TM^ software, importing the simulated diffraction patterns and the experimental Fourier transform into PhotoShop^TM^ software, applying transparency to the simulated diffraction patterns, and using each major reflection in the 3C pattern as an origin for a copy of the 6H pattern.

## Electronic supplementary material

Supplementary information
