# Supplementary material for: Irradiation-induced β to α SiC transformation at low temperature
Source: Sci Rep. 2017 Apr 26;7:1198. doi: 10.1038/s41598-017-01395-y (PMC5430638; doi:10.1038/s41598-017-01395-y)
Supplement: Supplementary file 1 — Supplementary information [file 41598_2017_1395_MOESM1_ESM.pdf]

Supplemental information to

Irradiation-induced β to α SiC transformation at low temperature

Chad M. Parish[1], Takaaki Koyanagi[1], Sosuke Kondo[2], and Yutai Katoh[1]
[1] Oak Ridge National Laboratory, Oak Ridge, TN 37831, USA
[2] Institute of Advanced Energy, Kyoto University, Uji, Kyoto, Japan 611-0011

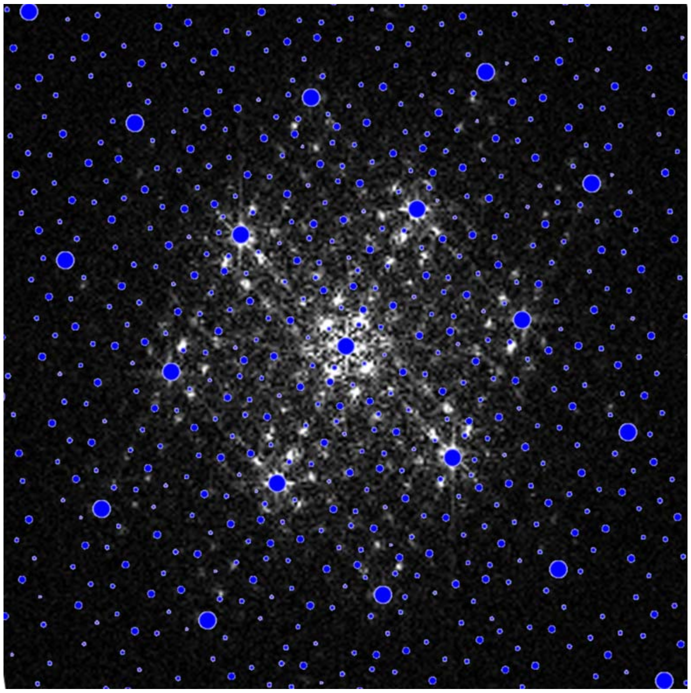

Supplemental Figure S1: Fourier transform from Figure 5(c), with superimposed double-diffraction pattern of <0001>$_{\alpha\text{-SiC}}$ // <101>$_{\beta\text{-SiC}}$ superimposed (blue).
